# Water deficit differentially modulates leaf photosynthesis and transpiration of fungus-tolerant *Muscadinia* x *Vitis* hybrids

**DOI:** 10.3389/fpls.2024.1405343

**Published:** 2024-05-16

**Authors:** Luciana Wilhelm de Almeida, Claudio Pastenes, Hernán Ojeda, Laurent Torregrosa, Anne Pellegrino

**Affiliations:** ^1^ UE Pech Rouge, Univ Montpellier, INRAE, Gruissan, France; ^2^ UMR LEPSE, Univ Montpellier, INRAE, CIRAD, Institut Agro Montpellier, Montpellier, France; ^3^ Departamento de Producción Agrícola, Facultad de Ciencias Agronómicas, Universidad de Chile, Santiago, Chile

**Keywords:** chlorophyll fluorescence, stomatal conductance, whole-plant transpiration, berry sugarless trait, grapevine

## Abstract

Screening for drought performance among novel fungi-tolerant grapevine genotypes is a key point to consider in semiarid regions where water scarcity is a common problem during fruit ripening period. It is therefore important to evaluate the genotypes’ responses at the level of carbon metabolism and water demand, under water deficit conditions. This study aimed to characterize leaf and plant water use efficiency (respectively named WUEi and WUEpl) of novel INRAE fungi-tolerant genotypes (including LowSugarBerry (LSB) genotypes), under mild and high-water deficit (WD) and to decipher the photosynthetic parameters leading to higher WUEi. For this purpose, experiments were conducted on potted plants during one season using a phenotyping platform. Two stabilized soil moisture capacity (SMC) conditions, corresponding to mild (SMC 0.6) and high (SMC 0.3) WD, were imposed from the onset of berry ripening until the physiological ripeness stage, which was defined as the point at which fruits reach their maximum solutes and water content. At the whole plant level, all genotypes increased WUEpl under high WD. The highest WUEpl was reached for 3176N, which displayed both a high rate of non-structural carbon accumulation in fruits due to high fruit-to-leaf ratio and low plant transpiration because of low total leaf area. However, when normalizing the fruit-to-leaf ratio among the genotypes, G14 reached the highest normalized WUEpl_n under high WD. At the leaf level, WUEi also increased under high WD, with the highest value attained for G14 and 3176N and the lowest value for Syrah. The higher WUEi values for all genotypes compared to Syrah were associated to higher levels of photosynthesis and changes in light-harvesting efficiency parameters (Φ_CO2_, qP and qN), while no clear trend was apparent when considering the photosynthetic biochemical parameters (Vcmax, Jmax). Finally, a positive correlation between leaf and plant WUE was observed regardless of genotypes. This study allowed us to classify grapevine genotypes based on their grapes primary metabolite accumulation and water consumption during the critical sugar-loading period. Additionally, the study highlighted the potential drought adaptation mechanism of the LSB genotypes.

## 1 Introduction

Grapevines are a prominent global fruit crop, largely cultivated in semi-arid regions with rainfed or under-deficit irrigation systems. Because of climate change, several viticultural regions are currently encountering more frequent and severe droughts (Santillán et al., 2020), with significant impacts on the vineyards’ resilience, grape yield and composition (Van Leeuwen et al., 2019; Santillán et al., 2020). With an emphasis on more eco-friendly wine and grape production that uses fewer chemical inputs in vineyards, challenges such as fungal diseases, including downy and powdery mildews, sums to this challenge. So, it becomes urgent to propose varietal innovations combining both tolerance to fungal diseases and water deficit (WD). Despite numerous breeding programs proposing new disease-tolerant hybrids (Yobrégat, 2018), there is still a lack of characterization of these genotypes’ response to abiotic factors, such as drought.

When screening for drought performance among grapevine genotypes, it is crucial to consider both the carbon metabolism and water demand responses to WD. The water use efficiency (WUE) has been widely recognized as a useful parameter (Hoover et al., 2023) when seeking for vineyard sustainability (Tomás et al., 2012), especially under adverse climate change conditions like drought (Andreu-Hayles et al., 2011; Gago et al., 2014). The WUE is defined as the balance between the carbon gain and water loss, and it can be measured at different scales, from leaf to ecosystem and at different time frames, from seconds to years (Medrano et al., 2015a; Hoover et al., 2023). At the leaf level, it is calculated as the ratio of net photosynthesis (An) to stomatal conductance (gs) (WUEi) or to transpiration (E) (WUEinst). At the plant level WUE can be assessed as the ratio of plant carbon gain to total transpired water (WUEpl) (Flexas et al., 2010; Hoover et al., 2023). Carbon isotopic discrimination analysis (δC^13^), in grapes, is an integrative indicator of WUEi during grape maturation, and might be considered as a proxy to plant WUE during ripening (Santesteban et al., 2015). To assess ecosystem WUE over longer time frames, indicators such as radial wood growth, δC^13^, and eddy covariance are commonly used (Tang et al., 2015; Medlyn et al., 2017; Gong et al., 2022).

High genotypic variability in grapevine WUEi under WD (Bota et al., 2001, Bota et al., 2016; Gutiérrez-Gamboa et al., 2019; Wilhelm de Almeida et al., 2023a) and on δC^13^ (Tomás et al., 2014; Bchir et al., 2016; Bota et al., 2016; Jacinto et al., 2023) has been reported, mostly associated with contrasting gs regulations (Tomás et al., 2014). Although all cultivars decrease stomatal conductance under WD, the sensitivity of the stomatal control under WD differs significantly. This variation has been adopted for genotype classification, distinguishing those tending to maintain stomata open at high levels of WD resulting in a decrease in leaf water potential (near anisohydric), from those characterized by stringent stomata control, thereby maintaining the leaf water potential at a high level (near isohydric) (Schultz, 1996, Schultz, 2000). However, this classification can vary, leading to controversial results for the same cultivar, mainly due to differences in growing conditions, the hydraulic conductance control on gs and the degree of WD (Martínez-Vilalta and Garcia-Forner, 2017; Hochberg et al., 2018).

Enhancing WUEi under WD can also be achieved by investigating genotypic regulations on photosynthesis (Medrano et al., 2002; Flexas et al., 2010; Gago et al., 2014; Mathias and Thomas, 2021). For instance, Mathias and Thomas, 2021 showed that the consistent increase in WUEi in several woody species across the globe were mainly related to stimulated An and not to gs reductions. Although stomatal limitations account for most of the An variation under WD, there are also non-stomatal limitations to consider, such as those linked to light harvesting efficiency and biochemical processes (Gago et al., 2014). Under no light limiting conditions, factors such as mesophyll conductance (gm), Rubisco (RuBP) activity and carboxylation efficiency (Vcmax), RuBP regeneration (maximum electron transport rate, Jmax) and the maximum rate of triose-P use (TPU), are all vital biochemical processes that play a major role on An rates (Flexas et al., 2016; Urban et al., 2017). Yet, it has been observed that WD negatively impacts Vcmax, Jmax and gm (Lawlor and Tezara, 2009; Villalobos-González et al., 2022), and that such impacts were genotype dependent in grapevine (Tomás et al., 2014; Villalobos-González et al., 2022).

It is important to have in mind that a higher WUEi under WD does not always translate into increased WUEpl. This is due to different factors impacting WUE at the leaf and plant level. For instance, WUEi is highly dependent on vapor pressure deficit (VPD), temperature and air CO_2_ concentration (Hatfield and Dold, 2019; Mathias and Thomas, 2021) because of their impact on gs (Zufferey et al., 2000; Prieto et al., 2010; Greer, 2017). At the plant level, pruning and canopy management play a key role on the leaf to fruit balance and organ microclimate, thus impacting the total carbon gain and water loss (Flexas et al., 2010; Prieto et al., 2012; Sexton et al., 2021). Previous studies reported a large variability of WUEpl among genotypes (Tomás et al., 2012). However, the expected drought-induced increase in this trait was not always observed (Poni et al., 2009; Tomás et al., 2012). When compared to WUEi and δC^13^ studies, there are much fewer studies focusing on grapevine WUEpl under WD. This might be due to the difficulties in quantifying total carbon gain and water losses at the whole plant level over the cropping season, and adds up to the complexity of factors contributing to WUEpl.

Furthermore, when coupling both WD and high light conditions, plants will tend to avoid excessive light-related damage, by activating photoprotective mechanisms such as energy dissipation as heat (Malnoë, 2018) and photorespiration (Villalobos-González et al., 2022). As for energy dissipation, two useful parameters associated to chlorophyll fluorescence can be used, qP which reflects the efficiency of photosystem II (PSII) in converting light energy into chemical energy during photosynthesis (proportion of PSII reaction centers open), and qN, which is related to thermal dissipation (Murchie and Lawson, 2013).

In this context, the present study aims to classify and characterize the performance of new fungal disease-tolerant varieties, including LSB genotypes which present lowered C demands in fruits (Bigard et al., 2022) under controlled conditions on potted plants under mild and high WD. It is hypothesized that novel fungi-tolerant genotypes, which include non *V. vinifera* genetic background could exhibit different responses to WD compared to *V. vinifera* cv. Syrah, a widely grown variety with well-documented performance characteristics. In particular, the WUE rates at leaf and plant levels were compared and the underlying impact of the photosynthetic machinery on the leaf WUE was addressed. We have worked with mild and high water deficit conditions, based on the real conditions of grapevine cultivation. Indeed, grapevines are grown worldwide under conditions of inherent water deficit, especially during the critical period from veraison to harvest, which coincides with hotter and drier conditions of summer. The simulation of these suboptimal water conditions in our study is essential for understanding grapevine responses to stress and adapting them for improved vineyard management.

## 2 Materials and methods

### 2.1 Overall plant material and growing conditions

The plant material consisted of five fungus disease-tolerant scions of grapevine, Floreal, 3176N, 3159B, G5 and G4, which are *V. vinifera* x *M. rotundifolia* derivative hybrids (Wilhelm de Almeida et al., 2023a), where the latter two display the LSB trait. The *V. vinifera* L. var. ‘Syrah’ was adopted as the control. All plants were three years old and grafted on 140 Ruggeri rootstock. Plants were installed in 9 L (0.19 m diameter x 0.4 m high) pots filled with a substrate composed of clay (170 kg m^-3^) and peat (50% of frozen black peat and 50% of white peat). Each plant was spur pruned to keep two proleptic axes. Thus, two annual shoots with all the secondary axes, and two to three clusters per plant were retained. A total of 10 plants per genotype were assessed, resulting in population size of 60 plants.

From budburst until the start of veraison (defined as 10% of softened berries) plants were cultivated outside and managed to avoid any mineral/water deficit or pest/disease development. Fertilization consisted of a nutritive solution composed of 2.2% nitrogen 1.6% P_2_O_5_, 6.4% K_2_O, 1.6% MgO and 3.2% SO_3_, with a proportion of nutritive solution to water of 0.2%. Two inputs of iron fertilization (1.1 g Fe per plant) were performed, at 15 and 30 days after budburst.

At veraison and until the conclusion of the experiment, plants were transferred into the Phenodyn phenotyping platform to impose the targeted water treatments (https://www6.montpellier.inrae.fr/lepse/Plateformes-de-phenotypage-M3P). Phenodyn is an automated greenhouse equipped with a set of climatic sensors that measure light intensity, relative humidity, air temperature and VPD every minute. Each plant is equipped with a connected scale continuously measuring weight changes and an automatic dripper able to add precise amounts of water based on predetermined soil water contents target (Sadok et al., 2007). During the ripening period, at 10 days after veraison (DAV) a third application of iron was made (1.1 g Fe per plant) and an input of 3 g of Osmocote per plant (0.5 g of N per plant). Relative humidity, air temperature and VPD were respectively 62%, 27 °C and 1.4 kPa during the day, and 75%, 21 °C and 0.7 kPa during the night. The photosynthetic photon flux density (PPFD) during the day (from 6:00 h to 19:00 h) was 997 μmol m^-2^ s^-1^ on average.

### 2.2 Water treatments

Before veraison plants were watered to meet climatic demand. They were irrigated twice a day (10h00 and 14h00) from budburst until flowering and five times a day (8h00, 10h00, 14h00, 18h00 and 21h00) from flowering until veraison. The duration of the watering period was 9 minutes with a flow rate of 2 L h^-1^, corresponding to 0.6 L day^-1^ from budburst to flowering and 1.5 L day^-1^ from flowering to veraison.

Once plants entered the phenotyping platform (from veraison to physiological ripeness), genotypes and water treatment (mild WD ‘M-WD’ and high WD ‘H-WD’) were randomized in a split-plot design with five blocks. Within each block, genotypes were randomly distributed in the main plot while the water treatment to the subplot. A total of 5 replicates per ‘genotype × water treatment’ were assessed (5 plants × 6 genotypes × 2 water treatment = 60). M-WD and H-WD corresponded to soil moisture capacity (SMC) of 60% and 30% (respectively), which were maintained according to pot weight target values by daily irrigations (up to four times a day 8h00, 13h00, 17h00 and 20h00). For each individual plant, the target weight used for irrigation was calculated as in (Coupel-Ledru et al., 2014) and it was defined based on a pre-experiment using 4 potted plants at the same stage of development and substrate to the plants under study. The pre-experiment consisted of watering the plants to field capacity (FC) and bagging the pots to prevent evaporation from the soil. The plants went on a drying period until permanent wilting point (WP) assessed from predawn water potential (Ψ_pd_) measurements. The pots were weighted on each day, at the same time, from FC to WP. The relationship between Ψ_pd_ and soil water content is shown in **Supplementary Figure S1**. These values were used as a reference to calculate the SMC (Equation 1).


(1) $$\text{SMC }=\;\left(\text{Soi}{\text{l}}_{\text{TW}}-\text{ Soi}{\text{l}}_{\text{W}-\text{WP}}\right)/\left(\text{Soi}{\text{l}}_{\text{W}-\text{FC}}-\text{ Soi}{\text{l}}_{\text{W}-\text{WP}}\right)$$

where Soil_TW_ corresponds to the total pot weight measured daily minus the total tare, Soil_W-WP_ and Soil_W-FC_ to the soil weight at permanent wilting point (2691 g on average) and at field capacity (3064 g on average), both determined during the pre-experiment and applied to all plants. The total tare corresponded to the sum of plant fresh weight (measured by image analysis at veraison, as described below) and the tutor weight (200 g).

### 2.3 Definition of fruit physiological ripeness stage and cluster biomass

Mean berry weight at veraison was obtained from the berry volume determined from the images (BV_VER_), using the parameters of a linear regression fitted at harvest between the measured mean berry weight and the image-based berry volume at harvest (BV_HAR_):


(2) $$\text{Mean berry weigh}{\text{t}}_{\text{VER or HAR}}\left(\text{g}\right)\;=\text{ B}{\text{V}}_{\text{VER or HAR}}\left(\text{c}{\text{m}}^{3}\right)\;*\;0.56441\;-\;0.42320$$

The biomass of clusters at veraison was then determined by multiplying the mean berry weight estimated from the pictures taken at veraison by the number of berries per plant counted at harvest.

At the conclusion of the experiment, all plants were weighted to record vegetative fresh biomass (shoots + leaves). The dry biomass of leaves (Leaf_DW_) and shoots (Shoot_DW_) were assessed after drying all samples for 15 days at 60°C. At this same stage, images of clusters were taken for fitting the relation between mean berry weight and the estimated berry volume (BV_HAR_) (Equation 2). The number of clusters, fresh fruit weight (F_FW_) and number of berries were directly assessed. Berry weight was determined by dividing the weight of all berries by the total number of berries per plant.

### 2.4 Total leaf area and canopy biomass

The vegetative biomass, total leaf area of individual plants were estimated through RGB image analysis at the onset of their entry into the platform at veraison (see below) or from direct measurements at the conclusion of the experiment. The images of all plants at veraison were acquired within the PhenoArch platform (Cabrera-Bosquet et al., 2016), hosted at M3P (Montpellier Plant Phenotyping Platforms, https://www6.montpellier.inrae.fr/lepse/Plateformes-de-phenotypage-M3P). Images were captured for each plant from 13 different angles, which included 12 side views with a 30° rotational difference, as well as one top view.

For vegetative biomass and total leaf area estimations at veraison, plant pixels were separated from the background using a combination of thresholding and random forest algorithms, following the methodology described by (Brichet et al., 2017) and converted into mm^2^ by calibrating camera positions using reference objects. Then, total plant leaf area and shoot fresh weight were determined from calibration curves established with multiple linear regression models. These models were constructed based on processed images taken in the 13 directions (grapevine database) against ground truth measurements of leaf area and fresh canopy biomass (excluding biomass of clusters) at different stages. The latter measurements (total leaf area and fresh canopy biomass) were taken at the conclusion of the experiment on plants subjected to M-WD and H-WD treatments.

The total leaf area at the end of the experiment was assessed in one plant for treatment using a planimeter (LI-3100C Area Meter, LiCor Biosciences GmbH, Bad Homburg, Germany), and then estimated for all plants by fitting a linear regression between total leaf area and leaves dry weight (Leaf_DW_) (Equation 3).


(3) $$\text{Total plant leaf area }\left(\text{c}{\text{m}}^{2}\right)\;=\;239.94\;*\text{ Lea}{\text{f}}_{\text{DW}}\left(\text{g}\right)\;-\;4674.56$$

In order to characterize plant balance, the ratio of total cluster fresh weight to leaf area (kg m^-2^) at harvest was calculated.

### 2.5 Variation of fruit carbon content during fruit ripening

To evaluate the distribution of carbon in the major metabolites of the grapevine fruit, at physiological ripeness each individual plant was harvested and the juice of all berries was extracted. Soluble sugars (SS = glucose + fructose), tartaric (H2T) and malic (H2M) acids were analyzed by high-performance liquid chromatography (HPLC) and UV detector as described in (Bigard et al., 2019). To estimate the soluble carbon content in fruits at veraison, it was considered a concentration of SS, H2T and H2M, for normal sugar level genotypes (3176N, 3159B, Floreal and Syrah) of 100, 120 and 210 mmol L^-1^, respectively, and for sugarless genotypes (G14 and G5), of 83, 96 and 258 mmol L^-1^, respectively (Bigard et al., 2022). The carbon equivalent conversion was done for SS, H2T and H2M, using MM of 180, 150, 134 g mol^-1^, and number of C of 6, 4 and 4, respectively as in (Wilhelm de Almeida et al., 2023b). Thus, the variation of fruit carbon content per plant (varC) during the ripening period was determined as follow (Equation 4):


(4) $$\begin{array}{c} \text{varC }=\;\left({\left({\text{C}}_{\text{SS}}+\;{\text{C}}_{\text{H}2\text{M}}+\;{\text{C}}_{\text{H}2\text{T}}\right)}_{\text{HAR}}\;*\;{\text{F}}_{\text{FW}-\text{HAR}}\right)\;-\;\\ {\left({\left({\text{C}}_{\text{SS}}+\;{\text{C}}_{\text{H}2\text{M}}+\;{\text{C}}_{\text{H}2\text{T}}\right)}_{\text{VER}}\;*\;{\text{F}}_{\text{FW}-\text{VER}}\right)}_{\;} \end{array}$$

### 2.6 Plant transpiration

Total plant transpiration over the ripening period (TR) was obtained from the area under the curve of the individual plant pot weight variations recorded every 15 minutes.

### 2.7 Gas exchange measurements

All gas exchange measurements were performed on one mature and exposed leaf per plant, using a LI-6800 Portable Photosynthesis System equipped with the Multiphase Flash Fluorometer and Chamber (LI- COR Inc., Lincoln, NE, USA). The environmental parameters in the chamber were settled with flow rate of 600 μmol s-1, photosynthetically active radiation (PAR) of 1500 μmol photons m^-2^ s^-1^, CO_2_ of 400 µmol mol-1, VPD of 1.8 kPa, and leaf temperature of 28°C for most measurements. Leaves were systematically acclimated to the chamber setting conditions for 5 minutes prior to the measurement described below.

#### 2.7.1 Net photosynthesis, stomatal conductance and leaf transpiration

The net photosynthesis (An) and stomatal conductance (gs) were measured three to four times after veraison and before physiological ripeness, approximately at 5, 10, 25 and 40 days after veraison (DAV), in 5 to 6 plants per treatment.

#### 2.7.2 Maximum rate for carboxylation and electron transport

The parameters of maximum rate for carboxylation (Vcmax) and maximum electron transport rate (Jmax) were calculated from photosynthesis response curves to varying intercellular CO_2_ concentrations. Response curves were assessed at 10 DAV on 5 plants per genotype. Those response curves were assessed with the dynamic assimilation technique, utilizing a continuous CO_2_ ramp rate of 160 µmol mol^-1^ min^-1^ and measurements recorded at each four seconds (Saathoff and Welles, 2021). The reference and sample infrared gas analyzers (IRGAs) were matched every 20 minutes. The dynamic assimilation technique program consists of a ramp down from 400 to 10 µmol mol^-1^ of CO_2_, which is followed one minute later by a ramp up from 10 to 1100 µmol mol^-1^ of CO_2_, at a rate of 160 µmol mol^-1^ of CO_2_. The data used to fit the photosynthesis response curve consisted of the ramp up phase. The parameters Vcmax and Jmax were estimated from the A-Ci fit from the R package ‘plantecophys’, using the default method of the *fitacis* () function (Duursma, 2015).

#### 2.7.3 Photosynthesis response to light intensity and chlorophyll fluorescence

Dark respiration (Rd) and photosynthesis response curve to PAR (An-PAR) were performed at 25 DAV on 4 plants per treatment.

To assess Rd, leaves were covered with foil paper and dark acclimated for 12 h prior to gas exchange and chlorophyll fluorescence measurements (Fm and Fo). Modifications on chamber environmental conditions were done regarding flow rate, set to 400 μmol s^-1^ and PAR set to 0 μmol photons m^-2^ s^-1^.

After assessing the dark-adapted parameters, leaves were acclimated at 1800 μmol photons m^-2^ s^-1^ and gas exchange measurements and chlorophyll fluorescence measurements (Fm’, Fo’ and Ft) were taken at three to five minutes intervals at decreasing PAR levels: 1800, 1500, 1200, 900, 700, 600, 500, 400, 300, 200, 100, 50 and 0 μmol photons m^-2^ s^-1^.

Photosynthesis response curve to PAR were then fitted with the non-rectangular hyperbola model as described in (Villalobos-González et al., 2022), using the *nls()* function and the selfstart package for this model, *SSnrh* from the *nlraa* package (Archontoulis and Miguez, 2015). The parameters of maximum photosynthetic rate (Asyn) and apparent quantum yield (Φ_CO2_) were then estimated.

The parameters of photochemical and non-photochemical fluorescence quenching, qN and qP respectively, were estimated for each level of light intensity as described in Villalobos-González et al. (2022).

### 2.8 Water use efficiency at the leaf and plant levels

Leaf water use efficiency was calculated as the ratio of An to gs (WUE_i_). Plant water use efficiency was calculated as the ratio of the fruit carbon (in major soluble components) variation during ripening in soluble sugars (SS), malic acid (H2M) and tartaric acid (H2T) (varC, Equation 4) to total transpired water from veraison to harvest (TR) (WUE_PL_, Equation 5). A normalized WUE (WUEpl_n) was also calculated considering a plant with a plant balance of 1 L of fruit to 1 m^2^ of leaf area, in order to buffer the effects of the variations in yield per plant and plant leaf area between the genotypes on WUEpl (Equation 6).


(5) $$\text{WU}{\text{E}}_{\text{PL}}\left(\text{g C }{\text{L}}^{-1}\right)\;=\text{ varC}:\text{ TR}$$

(6) $$\text{WU}{\text{E}}_{\text{PL}\_\text{n}}{\left(\text{g C }{\text{L}}^{-1}\right)}_{\;}=\text{ WUEpl }*\text{ Fruit}-\text{to}-\text{leaf ratio}$$

### 2.9 Statistical analysis

In order to account for the split-plot experimental design, the *lmer()* function was used to fit mixed effects models, where the fixed effects included blocks and the genotype’s interaction with water treatment, while the interaction of blocks and genotypes were considered as random effects. The average mean values of An, gs and WUEi per water treatment, M-WD and H-WD, corresponded to the average values per plant, when SMC ranged from 0.45 to 0.6 and from 0.15 to 0.3, respectively. The values used for qN and qP were those at 1200 PAR (maximum light intensity inside the platform). Multiple comparisons of means were performed using the *emmeans* and *multcomp* package, followed by pairwise comparisons with Bonferroni adjustment, with a significance level set at 0.05. To analyze the leaf and plant WUE relationship, Pearson correlations were assessed. The multivariate analysis (PCA) was conducted using FactoMiner package. In order to explore the contribution of variables to total plant transpiration (TR) and WUE (WUEpl), multiple linear regression was employed. The proportion explained by each variable considered in TR (gs, leaf area, sugar loading duration, varC) and in WUEpl (fruit to leaf ratio, TR, WUEi, varC) was calculated by dividing the sum of square by the total sum of square (η^2^). All graphical processing and statistical tests were performed using R studio software.

## 3 Results

### 3.1 Soil water content capacity, phenology and plant balance

The targeted SMC among water treatments were stable during the duration of the experiment, varying slightly among genotypes, with the average SMC of M-WD treatments ranging from 0.62 in G14, to 0.51 in G5 (**Table 1**). The H-WD showed significantly lower SMC values (*ca.* -47%), ranging from 0.33 in G14 to 0.27 in Floreal (p.value > 0.05). No interaction between treatments was observed (**Table 1**).

**Table 1:** Soil water content capacity (SMC), fruit to leaf ratio (kg m^-2^), carbon gain in fruit solubles solids per plant (varC) and total transpired water per plant, from veraison to harvest, in 5 fungus tolerant genotypes and Syrah under M-WD and H-WD treatments.

Variables		Syrah	3176N	3159B	Floreal	G14	G5
Soil moisture capacity (SMC)							
	M-WD	0.57 ± 0.03	0.53 ± 0.01	0.53 ± 0.02	0.54 ± 0.02	0.62 ± 0.02	0.51 ± 0.07
	H-WD	0.29 ± 0.01	0.28 ± 0.01	0.28 ± 0.02	0.27 ± 0.01	0.33 ± 0.02	0.28 ± 0.02
	Relative Diff. (%)	-48	-47	-48	-49	-46	-44
	G ***	*a*	*a*	*a*	*a*	*b*	*a*
	Treat ***						
	*block ns*						
	*G:Treat ns*						
Fruit to leaf ratio (kg m^-2^)							
	M-WD	0.58 ± 0.18	0.70 ± 0.28	0.18 ± 0.08	0.27 ± 0.13	0.20 ± 0.10	0.38 ± 0.17
	H-WD	0.49 ± 0.19	0.62 ± 0.14	0.17 ± 0.07	0.20 ± 0.08	0.16 ± 0.05	0.39 ± 0.17
	Relative Diff. (%)	-15	-11	-5	-25	-18	3
	G ***	*bc*	*c*	*a*	*a*	*a*	*ab*
	*Treat ns*						
	*block ns*						
	*G:Treat ns*						
Carbon gain in fruit solubles from veraison to harvest (varC) (g C per plant)							
	M-WD	26.8 ± 9.3	24.8 ± 3.5	9.8 ± 5.6	15.4 ± 7.2	12.3 ± 5.7	21.6 ± 6.2
	H-WD	20.5 ± 6.1	20.1 ± 3.6	9.7 ± 2.5	10.8 ± 3.1	7.7 ± 2.1	16.3 ± 4.7
	Relative Diff. (%)	-24	-19	-1	-30	-38	-24
	G ***	*c*	*c*	*ab*	*ab*	*a*	*bc*
	Treat **						
	*block ns*						
	*G:Treat ns*						
Total transpired water from veraison to harvest (TR) (L per plant)							
	M-WD	31.1 ± 3.7	27.3 ± 2.2	33.9 ± 2.3	29.3 ± 3.3	24.6 ± 1.7	26.7 ± 3.2
	H-WD	21.2 ± 2.4	17.8 ± 3.7	21.9 ± 2.7	20.2 ± 3.0	12.6 ± 3.5	17.7 ± 2.6
	Relative Diff. (%)	-32	-35	-35	-31	-49	-34
	G ***	*bc*	*ab*	*c*	*bc*	*a*	*ab*
	Treat ***						
	*block ns*						
	*G:Treat ns*						

Relative Diff. (%) was calculated as H-WD - M-WD/M-WD * 100. ‘G’, ‘Treat’ and ‘G: Treat’ correspond to genotype, water treatment and their interaction effects, respectively. ‘***’, ‘**’ and ‘*’ indicates significant differences at p ≤ 0.001, 0.01 and 0.05, respectively, and ‘ns’ indicates no-statistical significance.Different letters in the same row indicate statistical differences among genotypes regardless of irrigation treatment (Bonferroni adjustment).

Veraison started first in 3176N (DOY 181) and occurred lastly in G14, 19 days later (DOY 200) (**Table 2**). Sugar loading duration was also extreme for those two genotypes, ranging from 55 days in 3176N under H-WD vs 36 days in G14 under M-WD. Most genotypes showed longer durations (up to 10 days) to reach grape physiological ripeness in H-WD when compared to M-WD plants (**Table 2**). However, in G14 there were no differences on the sugar loading duration between water treatments, and Syrah was the only genotype with an opposite response, i.e. H-WD plants reached physiological ripeness 6 days earlier than M-WD (**Table 2**).

**Table 2:** Day of the year of veraison and physiological ripeness stage and sugar loading duration in 5 fungus tolerant genotypes and Syrah under M-WD and H-WD treatments.

	Veraison	Physiological ripeness stage		Sugar loading duration	
		M-WD	H-WD	M-WD	H-WD
Syrah	185	235	229	50	44
3176N	181	229	236	48	55
3159B	188	236	242	48	54
Floreal	188	230	235	42	47
G14	200	236	237	36	37
G5	191	235	241	44	54

Although the fruit fresh mass of the plants was adjusted at the beginning of the experiment, the remaining variations of total leaf area and yield components (berry number and berry weight) among the genotypes (**Supplementary Table S1**) led to contrasting plant balances (ratio of fresh fruit weight per unit of leaf area) at harvest (**Table 1**) between genotypes. The genotypes were divided into three main groups, the first conformed by Syrah and 3176N, displaying the highest number of berries and plant balance, with an average ratio of 0.60 kg m^-2^ irrespective of the water treatment. The second group was conformed by 3159B, G14 and Floreal, showing a lower number of berries and the lowest ratio of 0.20 kg m^-2^ on average (irrespective of the water treatment). The third group was conformed by G5, which displayed a similar berry number as the second group, but higher berry weight, thus resulting in intermediate plant balance value of 0.39 kg m^-2^ (**Table 1**).

### 3.2 Carbon gain and water loss at the plant level

#### 3.2.1 Total carbon gain as fruit soluble solids and total transpiration per plant

Variation in C gain (varC), i.e. the variation of the main sugars and organic acids (Equation 4) during ripening, was determined by both genotype and water treatment (**Table 1**). As observed for the fruit to leaf ratio, the C gain was higher for Syrah and 3176N than for Floreal, G14 and 3159B, with an average of 22.3 g C per plant and 11.0 g C per plant, respectively. G5, on the other hand, resulted in an intermediate value of 19 g C per plant. The varC values of the H-WD plants was 23% lower than that of M-HD, with no main differences between genotypes (**Table 1**). One exception was 3159B which showed stable varC regardless of water treatment (**Table 1**). Total plant transpiration during ripening (TR), under M-WD, ranged from 24.6 L in G14 to 33.9 L in 3159B, although their leaf areas were similar (**Table 1**; **SupplementaryTable S1**). Plants under H-WD treatment transpired 36% less of that observed in M-WD (10 L less, on average, over the ripening period).

#### 3.2.2 Observed and normalized values for plant water use efficiency

**Figure 1:**
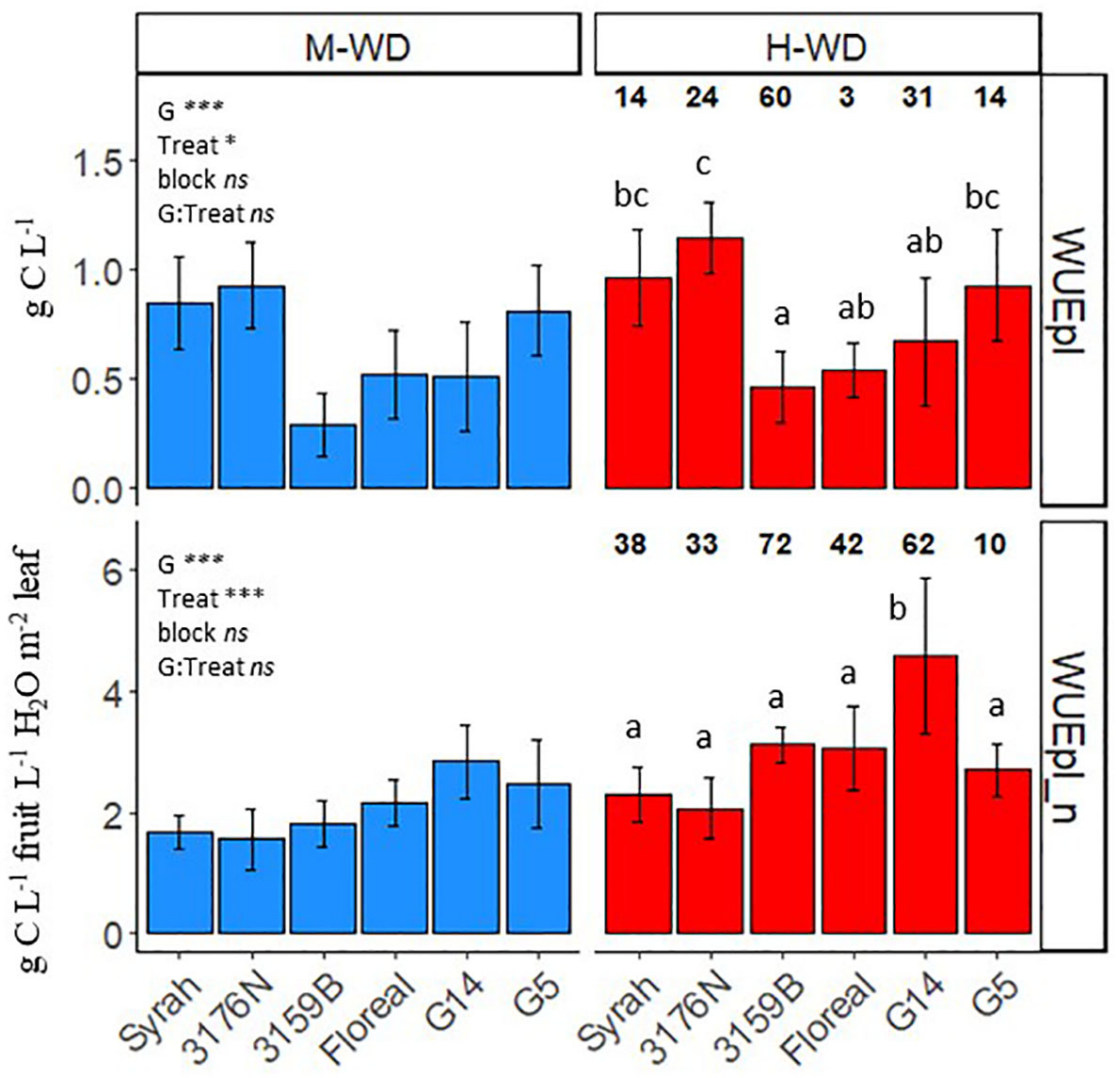
Means and standard deviations of observed (WUEpl) and normalized per fruit to leaf ratio (WUEpl_n) plant water use efficiency, from veraison to physiological ripeness in 5 fungus tolerant genotypes and Syrah under M-WD and H-WD treatments. Numbers on top indicate the relative difference (%) (calculated as H-WD - M-WD/M-WD * 100). Different letters indicate significant differences between genotypes averaging both water treatments. ‘G’, ‘Treat’ and ‘G: Treat’ stands for the genotype, water treatment and their interaction effects, respectively. ‘***’ and ‘*’ stands for 0.001, and 0.05 levels of significance and ‘ns’ to no-statistical significance.

The observed WUEpl values (**Figure 1**) varied according to the fruit to leaf area ratio (**Table 1**), with the highest being recorded for 3176N and the lowest for 3159B, with values of 1.03 g C L^-1^ of 0.37 g C L^-1^, respectively. In order to account for the phenotypic variations observed in both yield and leaf area per plant (**Supplementary Table S1**), the WUEpl was normalized by the plant balance (WUEpl_n, see Equation 5). This normalization is equivalent to calculating the grams of carbon gained per liter of transpired water for a plant displaying 1 L of fruit and 1 m^2^ of leaf area (**Figure 1**). The WUEpl_n was similar for all the genotypes, with an average value of 2.1 g C L^-1^ in M-WD, with the exception of G14, which reached a higher value of 2.8 g C L^-1^ (**Figure 1**). Although the variations of the fruit to leaf ratio were not significant between the water treatments (**Table 1**), the plant WUE increased in H-WD compared to M-WD by *ca*. 46% when normalized (WUEpl_n) *vsca*. 25% when non normalized (WUEpl) (**Figure 1**).

### 3.3 Leaf gas exchange response to water deficit

**Figure 2:**
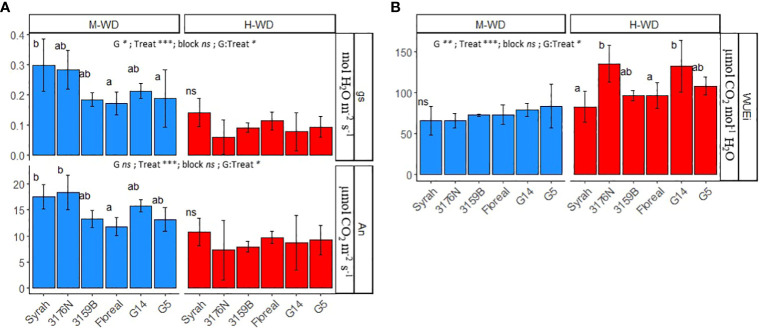
Means and standard deviations of gs, An **(A)** and WUEi **(B)** in 5 fungus tolerant genotypes and Syrah under M-WD and H-WD treatments. Different letters indicate significant differences between genotypes within water treatment. ‘G’, ‘Treat’ and ‘G: Treat’ stands for the genotype, water treatment and their interaction effects, respectively. ‘***’, ‘**’, ‘*’ stands for 0.001, 0.01, 0.05 levels of significance and ‘ns’ to no-statistical significance.

The average mean values of An, gs and WUEi were compared among genotypes under both M-WD and H-WD treatments (**Figure 2**). The values corresponded to the average values per plant, when SMCinst ranged from 0.45 to 0.6 and from 0.15 to 0.3, respectively. The WUEi is presented as a representative variable of leaf instantaneous WUE (WUEinst) due to the constant VPD throughout the experiment, which resulted in a high correlation between gs and E.

#### 3.3.1 Photosynthesis, stomatal conductance and leaf WUE responses to water deficit

All genotypes reduced the net photosynthesis and stomatal conductance, and increased WUEi from M-WD to H-WD (**Figure 2**). Differences on gs and An among genotypes were mostly observed under M-WD (**Figure 2A**), while on WUEi differences arose under H-WD (**Figure 2B**).

Under M-WD genotypes exhibited either comparable (3176N, 3159B, G14) or lower (Floreal and G5) gs and An values compared to Syrah (0.298 mol H_2_O m^-2^ s^-1^ and 17.5 μmol CO_2_ m^-2^ s^-1^, respectively). While under H-WD, all genotypes displayed similar average gs and An values of 0.095 mol H_2_O m^-2^ s^-1^ and 8.90 μmol CO_2_ m^-2^ s^-1^, respectively (p-value ≥ 0.05) (**Figure 2A**). The highest regulations on gs and An were displayed by 3176N, which showed a decrease of 79% and 60%, respectively when comparing M-WD and H-WD (**Supplementary Table S2**). Despite G14 showing a similar decrease in gs values, of 62%, it showed a similar reduction in An as that of Syrah, of 37% (**Supplementary Table S2**).

Consequently, there were consistent WUEi values among genotypes under M-WD conditions (averaging 73 μmol CO_2_ mol^-1^ H_2_O), but variations emerged under H-WD conditions, where those genotypes that showed the highest gs regulations, 3176N and G14, also exhibited the highest values of 96.3 μmol CO_2_ mol^-1^ H_2_O and 132.4 μmol CO_2_ mol^-1^ H_2_O, respectively (**Figure 2B**).

#### 3.3.2 Assessing photosynthesis parameters responses to water deficit

To better understand the limitations on photosynthetic parameters and water use efficiency under increasing drought, we compared the averages of the two water treatments for Φ_CO2_, Vcmax, Jmax, qP and qN.

**Figure 3:**
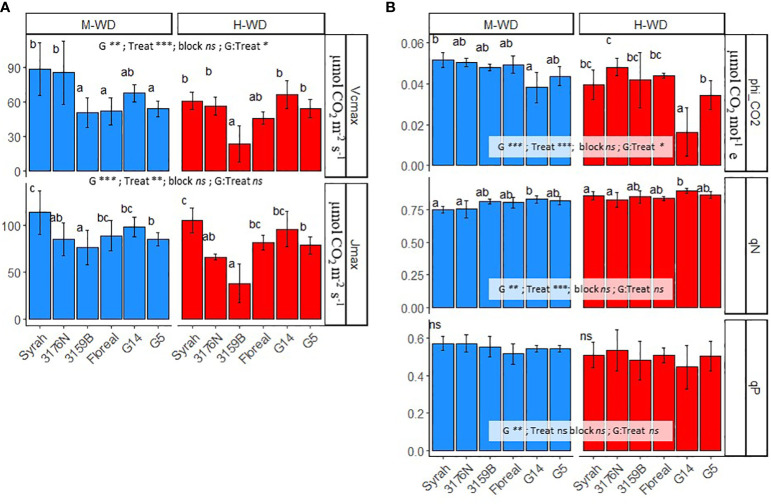
Means and standard deviations of Vcmax, Jmax **(A)** and of phi_CO_2_ (ΦCO_2_), qP and qN **(B)** in 5 fungus tolerant genotypes and Syrah under M-WD and H-WD treatments. Different letters indicate significant differences between genotypes within water treatment. ‘G’, ‘Treat’ and ‘G: Treat’ stands for the genotype, water treatment and their interaction effects, respectively. ‘***’, ‘**’, ‘*’ stands for 0.001, 0.01, 0.05 levels of significance and ‘ns’ to no-statistical significance. Values of qP and qN presented are at 1200 PAR.

Under M-WD, Vcmax values were either comparable (3176N and G14) or lower (3159B, Floreal and G5) than the values observed for Syrah. Genotypes showed different reduction rates when comparing H-WD to M-WD, where Syrah, 3176N and 3159B reduced Vcmax values of more than 30%, while Floreal, G14 and G5 were not significantly affected (**Figure 3A**). This led to more attenuated genotypic differences under H-WD, with only 3159B showing lower values, of 23.7 μmol CO_2_ m^-2^ s^-1^, than those displayed by Syrah, 61.1 μmol CO_2_ m^-2^ s^-1^ (**Figure 3A**). Differently, Jmax showed similar genotype ranking under both M-WD and H-WD, with genotypes showing values either similar (Floreal and G14) or lower (3176N, 3159B and G5) than Syrah. A general decrease in Jmax values of 16%, regardless of genotype, from M-WD to H-WD was also observed (**Figure 3A**).

In terms of Φ_CO2_ and qP, genotypes showed comparable values to Syrah, of 0.046 µmol CO_2_ mol^-1^ and 0.54, respectively, under both water treatments (**Figure 3B**). An exception was observed for Φ_CO2_ where G5 showed lower values than Syrah, on average 0.039 µmol CO_2_ mol^-1^ (regardless of water treatment). Yet, most genotypes showed similar qN values than those of Syrah, of 0.85 and 0.75 in M-WD and H-WD, respectively. Whereas G14 stood out showing higher qN of 0.83 and 0.89, in M-WD and H-WD, respectively (**Figure 3B**).

### 3.4 Overall genotype responses to water deficit

**Figure 4:**
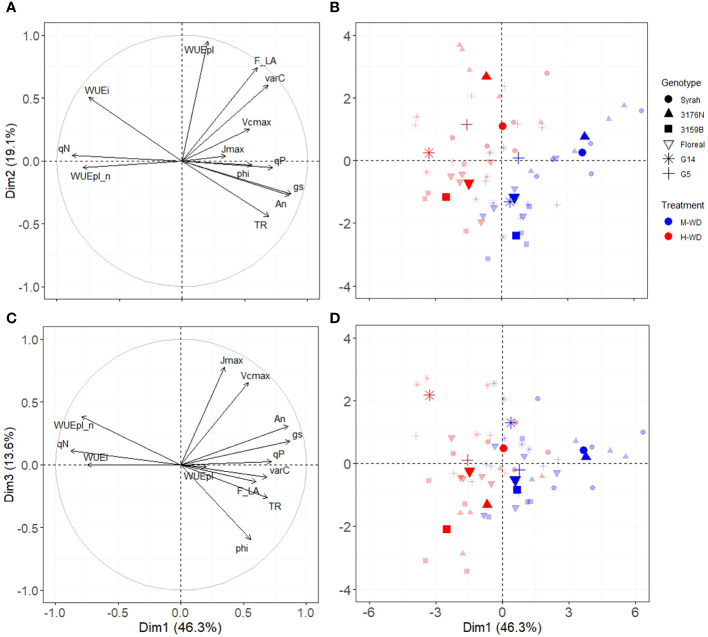
Principal components analysis in Dim1 and Dim2 **(A, B)** and in Dim1 and Dim3 **(C, D)** of genotypes leaf and plant performance under moderate (blue) and high (red) water deficit. Points in bold colors represent the mean by genotype and water treatment.

A principal component analysis was conducted using the leaf variables (An, gs, WUEi, Vcmax, Jmax, phi (Φ_CO2_), qP and qN) and plant variables [WUEpl, WUEpl_n, fruit to leaf ratio (F_LA), carbon gain in fruits (varC) and plant transpiration (TR)] (**Figure 4**). The PCA explained 78% of the variation, where the first, second and third dimensions (Dim1, Dim2 and Dim3) accounted for 46.3%, 19.1% and 13.6%, respectively (**Figure 4**). Dim1 distinctly separated both water treatments. An, gs, and qP were positively correlated with M-WD (right side), and qN, WUEi and WUEpl_n were related to H-WD (left side) (**Figures 4A, C**). Dim2 distinctly separated genotypes and it was mainly represented by WUEpl, and to a lesser extent by fruit to leaf ratio (F_LA), varC, WUEi and TR (**Figure 4A**). Syrah, 3176N and G5 were positively correlated to WUEpl and F_LA, and opposite to G14, 3159B and Floreal (**Figure 4B**). The genotypes classification on Dim2 was conserved at both water treatment levels, indicating similar values of WUEpl and fruit to leaf ratio, regardless of the water treatments (**Figure 4B**).

Dim3 was mainly related to leaf variables of Vcmax, Jmax, phi (Φ_CO2_) and An, and to a lesser extent to the plant variable of WUEpl_n (**Figure 4C**). On the right side (M-WD), Syrah and 3176N were related to high An and gs. While on the left side G14 was related to high WUEpl_n and qN and low phi and TR, while 3159B was related to low Jmax and Vcmax (**Figure 4D**).

### 3.5 Relationship between leaf and plant water use efficiency

**Figure 5:**
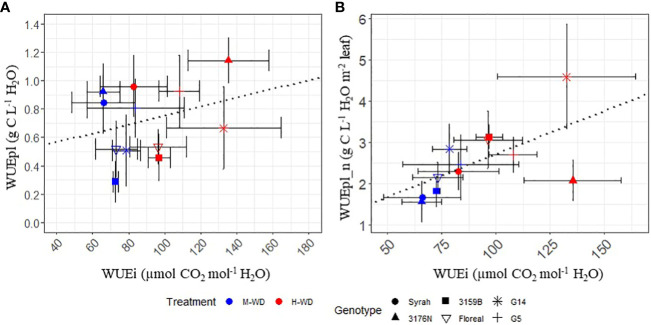
Linear relationship between leaf water use efficiency (WUEi) and plant WUE observed [WUEpl, **(A)**] and normalized per fruit to leaf ratio [WUEpl_n, **(B)**] in 5 fungus tolerant genotypes and Syrah, under M-WD (blue) and H-WD (red) treatments. Dotted lines represent the general correlation. WUEi values correspond to the average values of M-WD and H-WD, when SMC ranged from 0.45 to 0.6 and from 0.15 to 0.3, respectively.

The relationship between the leaf and plant WUE either observed (WUEpl) (**Figure 5A**) or normalized (WUEpl_n) (**Figure 5B**) was evaluated. A low correlation was found when analyzing WUEpl in function of WUEi (corr = 0.28, p.value = 0.04, **Figure 5A**).

After plant WUE was normalized, a positive correlation was observed between WUEi and WUEpl_n, indicating that greater WUEi was associated with a higher WUEpl_n (corr = 0.59, p.value< 0.001, **Figure 5B**). However, high differences were observed among the genotypes (**Supplementary Table S3**). Notably, G14 and 3176N showed the greatest deviation from the other genotypes. When comparing the increase in WUEpl_n between the two, G14 and 3176N showed respectively a higher and lower increase in WUEpl_n as WUEi increased (**Figure 5B**).

## 4 Discussion

In the present study, the response mechanisms of grapevines to contrasting WD levels imposed during the sugar loading into berry’s phase, was analyzed. The mild and high WD (0.6 and 0.3 of SMC) were chosen to represent common soil conditions faced by growers during the critical period of fruit ripening in many mediterranean regions.

High WD negatively affected most physiological variables related to carbon gain and water loss measured either at the leaf (An, gs) or plant level (TR, C accumulation in fruits), while increasing the leaf and plant WUE (WUEi, WUEpl). Although different signatures on the responses to WD were observed among the genotypes in leaf variables, the variations of fruit-to-leaf ratio also played a key cofactor role in those responses.

### 4.1 Leaf level regulations of water loss and carbon gain

Despite no differences in gs and An being observed among genotypes under H-WD conditions, G14 and 3176N exhibited the highest WUEi, while Syrah showed the lowest (**Figure 2**). Such variations were mostly related to a high gs reduction in the former genotypes, and a low reduction in the latter when comparing H-WD to M-WD (**Figure 2**; **Supplementary Table S2**). This less strict gs control of Syrah when compared to G14 and 3176N could be related to its well-reported near-anisohydric behavior under WD (Schultz, 2003).

A common leaf acclimation to WD is the regulation of gs to limit water loss, which subsequently reduces An rates and lead to increases in WUEi. Indeed, the variation in gs is the primary factor impacting both An and WUEi under WD (Tomás et al., 2014; Bota et al., 2016). For that, extensive research has been conducted on grapevine genetic variability in the regulation of gs under drought (Zufferey et al., 2000; Bota et al., 2001; Bota et al., 2016; Flexas et al., 2010; Prieto et al., 2010). In the present study, 3176N and Floreal exhibited respectively the highest and lowest gs reductions under H-WD (**Supplementary Table S2**). The control of stomata is associated with biochemical control, specifically abscisic acid, and hydraulic signaling, involving aquaporin proteins (Gambetta et al., 2017; Hasan et al., 2021). All of which might influence the differences in gs regulations between varieties (Coupel-Ledru et al., 2017; Shelden et al., 2017). Beyond the regulatory mechanisms influencing gs, the consistently lower gs values observed for 3159B, Floreal, G14, and G5, particularly under M-WD conditions and in comparison to Syrah, may be attributed to differences in stomatal anatomy and density among genotypes. These traits were observed to vary among different rice (Chen et al., 2020; Pitaloka et al., 2022), and soybean (Sakoda et al., 2019) varieties. However, environmental conditions can also influence these traits (Zhang et al., 2012). For instance, elevated temperature have been shown to lead to larger stomatas in grapevine (Sadras et al., 2012), while previous studies by Bota et al. (2016) have highlighted substantial genetic variability in gs values even under non-water deficit conditions.

Although this study confirms an important variability in such responses (gs, An, WUEi) among these novel genotypes, when comparing the genotype ranking in field conditions (Wilhelm de Almeida et al., 2023a) to the current controlled potted-vines experiment, contrasting results were found. In the preceding research 3159B, G5 and G14 displayed higher An reductions as WD increased, while Floreal and 3176N had similar An regulations to Syrah. The WUEi was similarly discordant, with Syrah and Floreal showing the highest increase in WUEi as WD progressed (Wilhelm de Almeida et al., 2023a).

Similar contrasting classifications between field and potted vines experiments were also reported by (Buesa et al., 2022), when studying leaf WUEi of different clones of *Vitis vinifera* ‘Grenache’ responses to WD. The sensitivity of genotypic gs response to drought changes in response to interactions between the genotypes and the environment, that includes the scion and rootstock pairs, the weather and microclimatic conditions (VPD, temperature, wind and light) and the degree of soil WD (Martínez-Vilalta and Garcia-Forner, 2017; Hochberg et al., 2018). The phenotyping platform maintains relatively constant conditions compared to open field conditions, with little variation in air VPD and temperature, low light intensities, and no wind. Additionally, soil conditions are highly contrasted as well, as potted plants are subjected to very dry soils with fluctuating conductivities and rapid wet and dry cycles, imposed by irrigation cycles. It is important to note that plants in the field might be more resilient, due to their higher reserve pool when compared to potted plants. This tight dependency to the plant’s environment can explain the lack of stability in genotype classification between studies performed in greenhouse and open-field.

### 4.2 Non-stomatal limitation of photosynthesis efficiency under water deficit

To account for the impact of photosynthetic properties on WUEi as WD progressed, we analyzed the biochemical (Vcmax and Jmax) and light-harvesting efficiency (Φ_CO2_, qP and qN) factors among genotypes (**Figure 3**). All parameters decrease under H-WD compared to M-WD, at different levels depending on variables, with exception to qN. At M-WD all genotypes exhibited a rather cohesive grouping when comparing the photosynthetic variables functioning, while at H-WD, a more scattered distribution of genotypes was observed in the PCA. This observed dispersion within lower water availability implies a heightened level of differentiation among genotypes, suggesting that the WD accentuated the inherent disparities between them.

Under H-WD the biochemical parameters Vcmax and Jmax both exhibited reductions of 17% and 16%, respectively (regardless of genotype). This observation aligns with their typical correlation, as high levels of carboxylation often require elevated reductive power (Manter and Kerrigan, 2004; Walker et al., 2014). The high decrease of Vcmax and Jmax under H-WD in 3159B suggests that plants may have modulated their carboxylation rate, potentially reducing enzyme activity when its substrate, i.e. CO_2_ is less available. In a previous study (Bota et al., 2004) it was observed that the content or activity of RuBP was reduced under high WD, after a 50% reduction in An.

It is important to note that in G14, G5, and Floreal, the biochemical process Vcmax was not negatively affected by H-WD, despite the fact that the two former genotypes displayed higher or similar reductions in gs and An compared to Syrah. This lack of effect could be attributed to the lower An when compared to those observed by Bota et al. (2004), but it could also be indicative of adaptive mechanisms in LSB genotypes that enable them to maintain Vcmax even under H-WD conditions. In the case of G14, this may have resulted in higher An and/or WUEi levels under H-WD when compared to Syrah. Nevertheless, other factors, such as a reduction in mesophyll conductance under WD (Tomás et al., 2014; Perdomo et al., 2017; Urban et al., 2017), may also contribute to a reduction in CO_2_ availability in chloroplasts (Galmés et al., 2011), resulting in a decrease in both Vcmax and Jmax.

Thermal dissipation is an important photoprotective mechanism activated in plants under environmental stress (Malnoë, 2018). Indeed, a negative relationship between gs and qN among genotypes was observed in the PCA (**Figure 4**), indicating that all genotypes increased heat dissipation under H-WD. Interestingly, G14 displayed a higher qN but similar qP than Syrah, suggesting a higher capacity in dissipating excess energy in the form of heat while maintaining a comparable maintenance of light conversion into chemical energy. Another photoprotective strategy adopted by C3 plants involves photorespiration (Kozaki and Takeba, 1996; Guan et al., 2004; Villalobos-González et al., 2022), which was proposed to be especially noticeable in genotypes with high sensitivity of stomatal regulation (near-isohydric behavior) (Villalobos-González et al., 2022). This might suggest that 3176N, with the highest regulation of gs but low qN, may have relied more on photorespiration.

Notably, the highest Φ_CO2_ observed for 3176N (**Figure 3B**) could be an interesting trait to enhance An under WD and low light conditions. This is particularly relevant in complex canopies such as in grapevines, where leaves are often shaded (in denser canopies) or subjected to intraday variations of light environments (in less-dense canopies) (Escalona et al., 2003; Prieto et al., 2012). Investigating the rapidity of gs responses would be a crucial point to better understand the contribution of this trait on C gain and WUEi under fluctuating light (Qu et al., 2016; Faralli et al., 2019).

### 4.3 Leaf and whole plant feedbacks under water deficit

In spite of crop load management among plants at the onset of the experiment, the fruit-to-leaf ratio was 3-folds higher in the present study for Syrah and 3176N compared to all other genotypes, regardless of the water treatments, while LSB genotypes showed either similar (G5) or lower (G14) ratios when compared to Syrah. Variations in fruit-to-leaf ratio were mostly related to variations in total leaf area (**Supplementary Table S1**). The higher ratio for Syrah and 3176N was a result of low leaf area associated to high yield. The high yield for these genotypes might be a result of genetic expression, as these two genotypes were also characterized as highly productive in field phenotyping experiments (Wilhelm de Almeida et al., 2023a). Genetic variability within grapevine yield formation was previously observed in regards to bunch number per shoot (Grzeskowiak et al., 2013), number of inflorescence per flower and in fruit set rate (Ibáñez et al., 2019).

Ultimately, a high influence of the fruit-to-leaf ratio on C accumulation was observed, where genotypes with higher ratios also showed a higher carbon gain in fruits (**Figures 4A, B**). As the ratio was consistently below 1 kg m^-2^ across all genotypes and water treatments, no trophic impediment in fruit maturation is expected. This can be due to a proper balance between sink and source activities (Kliewer and Dokoozlian, 2005), or yet due to the fact that as the sink force was very low for some genotypes, the expected source limitation due to WD was not enough to impair sugar accumulation (Intrigliolo and Castel, 2011). However, the longer sugar loading for H-WD compared to M-WD reflected an insufficient C gain in fruits. The fruit-to-leaf ratio not only influenced the C accumulation in fruits and WUEpl, but also An rates. For instance, the two genotypes with the highest fruit-to-leaf ratio, Syrah and 3176N, also exhibited the highest An rates. This could be attributed to a feedback response from sink (fruits) to source (leaf) organs. Feedback mechanisms between sink (crop load) and source activities (An rates) within the plant system have been reported in many fruit crops including apple (Pallas et al., 2018), peach (Wang et al., 2022) and grapevine (Rossouw et al., 2017; Martínez-Lüscher and Kurtural, 2021; Faralli et al., 2022). Conversely, plants of G5, despite having a similar fruit-to-leaf ratio to Syrah, did not exhibit increased An rates. This discrepancy might be associated with its LSB trait, indicating a lower carbon demand in the fruits, thereby influencing the assimilate allocation despite comparable ratios. Furthermore, plant balance was demonstrated to alter C reserves and mobilization in grapevines, further emphasizing its intricate and pivotal role in plant performance (Holzapfel and Smith, 2012; Hernández-Montes et al., 2022).

It is important to notice that when normalizing fruit-to-leaf ratio among genotypes, the levelling process aligns all plants based on those with lower productivity or vegetative expression. Consequently, this approach might suggest a skewed sense of comfort, favoring highly productive or vegetative genotypes like 3176N and 3159B (respectively) over inherently less fertile or vegetative ones such as Floreal and G5 (respectively). This also raises questions when considering genotypes inherently characterized by lower sugar demands in fruits, such as the LSB genotypes G14 and G5 (Bigard et al., 2022). When studying fleshy fruits of genotypes that decouple water and sugar demands, the definition of yield per se presents a direct challenge. Yield can be defined as fresh weight, which is mainly related to water demand, or as biomass, which is mostly linked to C demand. From a physiological perspective, this implies that genotypes should be normalized based on either water or C demand. If we consider yield in terms of fresh weight, i.e. in relation to the volume of fruit and therefore water, these genotypes that require less C, would theoretically be less reliant on photosynthesis during the sugar loading period. This suggests that they may exhibit lower photosynthetic rates. However, the photosynthetic rate did not appear to be particularly lower in the LSB genotypes. In addition, previous studies have proposed a relationship between high photosynthetic activity and C export to roots (Escalona et al., 2012; Hernández-Montes et al., 2022). This might imply that the G14 and G5 genotypes would have more C available to allocate to other plant sinks, such as reserves. A genotype-dependent response in C allocation to the root system was previously observed when comparing Tempranillo and Grenache under WD conditions, which was mainly accounted by their differences in C respiratory losses (Hernández-Montes et al., 2022). These characteristics could imply a superior performance of these LSB genotypes in facing WD conditions.

The total transpired water from veraison to harvest also varied among the genotypes and water treatments, due, at least partly to the fluctuations of total leaf area and of the duration of sugar loading (**Supplementary Figure S2**). Under M-WD, the high transpiration observed for 3159B was backed up by the highest total leaf area (**Supplementary Table S1**) and sugar loading durations (48 days) (**Table 2**). Syrah, which showed similar high transpiration, also showed the longest ripening period (50 days) although displaying one of the lowest total leaf areas. In contrast, G14 exhibited high vegetative development, but had one of the lowest transpiration rates due to its short sugar loading duration (36 days). Under H-WD, total transpired water per plant was reduced by *ca.* -36% compared to M-WD, in spite of a longer ripening duration for all genotypes except Syrah (up to 10 days). The reduction of transpiration is a recognized water conservation strategy in plants and it is ultimately linked to leaf area and gs regulations (Simonneau et al., 2017), indeed gs and TR were closely related in the PCA (**Figure 4A**). In the present study, total leaf area and gs were reduced under H-WD by about respectively 15% and 57%. Such low reduction of leaf area can be explained by the late onset of WD treatments (starting at veraison).

Although other factors such as boundary layer conductance, leaf cuticular conductance, stomatal density and size were shown to influence plant transpiration, of various plant species, including poplar (Grünhofer et al., 2022), grapevines (Konlechner and Sauer, 2016), rice (Chen et al., 2020; Pitaloka et al., 2022), and soybeans (Sakoda et al., 2019), it is likely that these variables accounted for variations between genotypes rather than differences arising from water treatments. Indeed, the boundary layer conductance was conserved between treatments inside the platform, due to its rather constant and controlled environmental conditions. In addition, the effects of drought on these traits are commonly established during early stages of leaf development (Bi et al., 2017; Bertolino et al., 2019), a period when all plants in our study experienced uniform, optimal conditions of light, water, and nutrients.

As the C accumulation in fruits was less reduced than plant transpiration under H-WD, WUEpl was promoted for all genotypes. However, the differences of WUEpl among the genotypes mainly resulted from the variations in the fruit-to-leaf ratio (**Supplementary Figure S3**). Yet, when normalized the WUEpl_n most genotypes showed comparable values to Syrah, despite showing lower plant transpiration. One exception was observed for the genotype G14 which showed the highest WUEpl_n (**Figure 1**). This suggests that under water-limiting situations, all fungi-tolerant genotypes tended to regulate their water loss over the ripening period more efficiently than Syrah, but G14 clearly stood out with a higher WUE. Furthermore, a lack of consideration for critical variables, including plant respiration and night transpiration, when assessing WUEi may contribute to observed discrepancies (Escalona et al., 2012; Medrano et al., 2015b; Coupel-Ledru et al., 2016). Such factors represent significant sources of carbon and water loss at the plant level. Studies have estimated that fruit carbon respiration alone accounts for approximately 18% of total leaf assimilated carbon (Hernández-Montes et al., 2022). Similarly, night transpiration can contribute up to 30% of daily water loss, particularly under dry conditions (Coupel-Ledru et al., 2016), with both factors demonstrating variability among grapevine genotypes. It is important to acknowledge that despite the normalization process allowing for a balanced comparison between genotypes at similar fruit-to-leaf ratios, i.e. considering a plant with 1 L of fruit and 1 m² of leaf area, it may introduce bias by assuming linearity in genotypic responses regardless of variations in the crop load. In addition, it do not considering the variations in C demand in genotypes presenting the LSB trait.

## 5 Conclusions

Physiological and biochemical responses of gas exchange related parameters varied depending on the genotype, highlighting the intricate relationship between genotypic traits and environmental conditions.

The fruit-to-leaf ratio emerged as a key determinant influencing C accumulation in fruits and WUEpl. Genotypes with higher fruit-to-leaf ratios demonstrated higher C gains in fruits and An rates, highlighting the role of sink-source interactions. Despite differences in responses under varying WD conditions, WUEpl was promoted for all genotypes due to reduced plant transpiration, with the LSB genotype, G14, exhibiting the highest normalized WUEpl.

When compared to Syrah, most genotypes displayed either equal or superior WUE at leaf and plant level. Two genotypes should be highlighted, 3176N and G14 due to their higher WUEi and different regulations in Φ_CO2_ in the former and in Vcmax and qN in the latter. Furthermore, the genotype-dependent correlation between leaf-level and whole-plant WUE emphasizes the need to further explore the significance of factors such as fruit-to-leaf ratio, canopy and root structure, plant respiration, and night transpiration in influencing overall WUE.

## Data availability statement

The raw data supporting the conclusions of this article will be made available by the authors, without undue reservation.

## Author contributions

LW: Conceptualization, Data curation, Formal analysis, Investigation, Methodology, Project administration, Software, Validation, Visualization, Writing – original draft, Writing – review & editing. CP: Conceptualization, Methodology, Resources, Supervision, Visualization, Writing – review & editing. HO: Conceptualization, Funding acquisition, Supervision, Writing – review & editing. LT: Conceptualization, Funding acquisition, Methodology, Project administration, Resources, Supervision, Validation, Visualization, Writing – review & editing. AP: Conceptualization, Funding acquisition, Investigation, Methodology, Project administration, Resources, Supervision, Validation, Visualization, Writing – review & editing.
