# Supplementary material for: Water deficit differentially modulates leaf photosynthesis and transpiration of fungus-tolerant Muscadinia x Vitis hybrids
Source: Front Plant Sci. 2024 May 16;15:1405343. doi: 10.3389/fpls.2024.1405343 (PMC11137165; doi:10.3389/fpls.2024.1405343)
Supplement: Supplementary file 1 [file DataSheet_1.docx]

## **Supplementary data for**

**Water deficit differentially modulates leaf photosynthesis and transpiration of fungus-tolerant Muscadinia x Vitis hybrids**

Luciana Wilhelm de Almeida ^1, 2^, Cláudio Pastenes ^3^, Hernán Ojeda ^1^, Laurent Torregrosa^1,2^ and Anne Pellegrino^2 *^

^1^ Unité Expérimentale INRAE de Pech Rouge, INRAE, 11430 Gruissan, France

^2^ UMR LEPSE, Univ Montpellier, INRAE, CIRAD, Institut Agro Montpellier, 2, place P. Viala, 34060 Montpellier Cedex, France

^3^ Facultad de Ciencias Agronómicas, Universidad de Chile, Santiago 8820808, Chile

*Corresponding author: anne.pellegrino@supagro.fr

E-mail address:

Tel:

2 Place Pierre Viala

34060 Montpellier Cedex 2

France

**Table S1.** Plant total leaf area, number of berries per plant and mean berry weight at harvest, in 5 fungus tolerant genotypes and Syrah under M-WD and H-WD treatments, Montpellier - FR, 2022.

| Variables |  | Syrah | 3176N | 3159B | Floreal | G14 | G5 |
| --- | --- | --- | --- | --- | --- | --- | --- |
| Total leaf area (m²) | | | | | | | |
|  | M-WD | 0.70 ± 0.17 | 0.69 ± 0.19 | 1.27 ± 0.09 | 0.98 ± 0.18 | 1.14 ± 0.21 | 1.14 ± 0.37 |
|  | H-WD | 0.63 ± 0.07 | 0.61 ± 0.15 | 1.13 ± 0.18 | 0.83 ± 0.16 | 1.04 ± 0.17 | 0.78 ± 0.14 |
|  | Relative Diff. (%) | -10 | -11 | -11 | -15 | -15 | -9 |
|  | *G **** | *a* | *a* | *b* | *ab* | *b* | *ab* |
|  | *Treat *** |  |  |  |  |  |  |
|  | *block ns* |  |  |  |  |  |  |
|  | *G :Treat ns* |  |  |  |  |  |  |
| Total number of berries | | | | | | | |
|  | M-WD | 310 ± 76 | 252 ± 64 | 167 ± 61 | 157 ± 64 | 148 ± 60 | 168 ± 38 |
|  | H-WD | 238 ± 66 | 224 ± 22 | 125 ± 24 | 106 ± 39 | 134 ± 17 | 140 ± 47 |
|  | Relative Diff. (%) | -23 | -11 | -25 | -32 | -9 | -17 |
|  | *G **** | *c* | *bc* | *ab* | *a* | *a* | *ab* |
|  | *Treat *** |  |  |  |  |  |  |
|  | *block ns* |  |  |  |  |  |  |
|  | *G :Treat ns* |  |  |  |  |  |  |
| Mean berry weight (g) | | | | | | | |
|  | M-WD | 1.28 ± 0.24 | 1.80 ± 0.18 | 1.35 ± 0.21 | 1.62 ± 0.08 | 1.48 ± 0.35 | 2.34 ± 0.25 |
|  | H-WD | 1.28 ± 0.26 | 1.66 ± 0.24 | 1.50 ± 0.14 | 1.58 ± 0.25 | 1.24 ± 0.15 | 2.12 ± 0.22 |
|  | Relative Diff. (%) | 0 | -8 | 11 | -2 | -16 | -9 |
|  | *G **** | *a* | *b* | *ab* | *ab* | *ab* | *c* |
|  | *Treat ns* |  |  |  |  |  |  |
|  | *block ns* |  |  |  |  |  |  |
|  | *G :Treat ns* |  |  |  |  |  |  |

**Table S2.** Means and standard deviations of gs, An, WUEi, Jmax, Vcmax, Φ_CO2_, qP and qN in 5 fungus tolerant genotypes and Syrah under M-WD and H-WD treatments. Different letters indicate significant differences between genotypes within water treatment. ‘ns’ indicates no-statistical significance. ‘*’ and ‘ns’ under each genotype indicate significance or no-statistical significance, respectively, of water treatment within each genotype.

| Variables |  | Syrah | 3176N | 3159B | Floreal | G14 | G5 |
| --- | --- | --- | --- | --- | --- | --- | --- |
| gs (mol H_2_O m^-2^ s^-1^) | | | | | | | |
|  | M-WD | 0.298 ^b^ ± 0.09 | 0.281 ^ab^ ± 0.06 | 0.188 ^ab^ ± 0.02 | 0.171 ^a^ ± 0.04 | 0.213 ^ab^ ± 0.02 | 0.188 ^a^ ± 0.10 |
|  | H-WD | 0.141 ^ns^ ± 0.05 | 0.060 ± 0.06 | 0.084 ± 0.02 | 0.114 ± 0.03 | 0.081 ± 0.06 | 0.093 ± 0.03 |
|  | Relative Diff. (%) | -53 | -79 | -55 | -33 | -62 | -51 |
|  | *G ** | *b* | *ab* | *a* | *ab* | *ab* | *a* |
|  | *Treat **** |  |  |  |  |  |  |
|  | *Block ns* |  |  |  |  |  |  |
|  | *G :Treat ** | *** | *** | *** | *ns* | *** | *** |
| An (µmol CO_2_ m^-2^ s^-1^) | |  |  |  |  |  |  |
|  | M-WD | 17.5 ^b^ ± 2.4 | 18.4 ^b^ ± 3.4 | 13.4 ^ab^ ± 1.7 | 11.8 ^a^ ± 1.7 | 15.8 ^ab^ ± 1.2 | 13.2 ^ab^ ± 2.3 |
|  | H-WD | 10.8 ^ns^ ± 2.7 | 7.3 ± 5.7 | 7.4 ± 1.1 | 9.8 ± 1.2 | 10.0 ± 5.2 | 9.2 ± 2.8 |
|  | Relative Diff. (%) | -38 | -60 | -45 | -17 | -37 | -30 |
|  | *G ns* |  |  |  |  |  |  |
|  | *Treat **** |  |  |  |  |  |  |
|  | *block ns* |  |  |  |  |  |  |
|  | *G :Treat ** | *** | *** | *** | *ns* | *** | *** |
| WUEi (µmol CO_2_ mol^-1^ H_2_O) | |  |  |  |  |  |  |
|  | M-WD | 66.0 ^ns^ ± 17.6 | 65.4 ± 9.1 | 71.1 ± 1.5 | 73.1 ± 11.6 | 78.6 ± 7.9 | 83.8 ± 26.8 |
|  | H-WD | 82.8 ^a^ ± 18.7 | 135.4 ^b^ ± 22.4 | 99.5 ^ab^ ± 6.2 | 96.3 ^a^ ± 15.8 | 132.4 ^b^ ± 31.8 | 108.0 ^ab^ ± 11.1 |
|  | Relative Diff. (%) | 25 | 103 | 40 | 32 | 68 | 29 |
|  | *G *** | *a* | *ab* | *ab* | *ab* | *b* | *ab* |
|  | *Treat **** |  |  |  |  |  |  |
|  | *block ns* |  |  |  |  |  |  |
|  | *G :Treat ** | *ns* | *** | *** | *** | *** | *** |
| Jmax (µmol CO_2_ m^-2^ s^-1^) | |  |  |  |  |  |  |
|  | M-WD | 114.2 ± 23.2 | 86.5 ± 17.6 | 75.4 ± 18.2 | 88.9 ± 16.2 | 98.3 ± 10.4 | 84.9 ± 6.8 |
|  | H-WD | 104.5 ± 13.2 | 62.7 ± 3.1 | 37.4 ± 20.3 | 81.4 ± 7.8 | 95.8 ± 18.7 | 78.7 ± 9.2 |
|  | Relative Diff. (%) | -7 | -23 | -51 | -13 | -3 | -9 |
|  | *G **** | *c* | *ab* | *a* | *bc* | *bc* | *b* |
|  | *Treat *** |  |  |  |  |  |  |
|  | *block ns* |  |  |  |  |  |  |
|  | *G :Treat ns* |  |  |  |  |  |  |
| Vcmax (µmol CO_2_ m^-2^ s^-1^) | | | | | | | |
|  | M-WD | 87.2 ^b^ ± 22.7 | 86.4 ^b^ ± 27.5 | 52.6 ^a^ ± 12.9 | 52.1 ^a^ ± 12.0 | 67.9 ^ab^ ± 7.5 | 54.2 ^a^ ± 6.6 |
|  | H-WD | 59.6 ^b^ ± 7.4 | 63.8 ^b^ ± 7.8 | 22.7 ^a^ ± 15.8 | 46.0 ^ab^ ± 5.2 | 66.8 ^b^ ± 12.2 | 54.3 ^b^ ± 7.8 |
|  | Relative Diff. (%) | -31 | -34 | -54 | -16 | -2 | -4 |
|  | *G *** | *bc* | *c* | *a* | *ab* | *bc* | *abc* |
|  | *Treat **** |  |  |  |  |  |  |
|  | *block ns* |  |  |  |  |  |  |
|  | *G :Treat ** | *** | *** | *** | *ns* | *ns* | *ns* |
| Φ x 10 ^-3^ (µmol CO_2_ mol ^-1^ photons) | | | | | | | |
|  | M-WD | 51.8 ^ns^ ± 3.6 | 50.5 ± 2.2 | 48.6 ± 1.7 | 47.6 ± 4.1 | 38.3 ± 7.4 | 44.9 ± 4.4 |
|  | H-WD | 39.7 ^b^ ± 7.3 | 55.4 ^c^ ± 4.3 | 41.9 ^bc^ ± 13.5 | 44.0 ^bc^ ± 1.3 | 16.3 ^a^ ± 11.9 | 34.5 ^b^ ± 7.1 |
|  | Relative Diff. (%) | -23 | 10 | -14 | -8 | -57 | -23 |
|  | *G **** | *bc* | *c* | *bc* | *bc* | *a* | *ab* |
|  | *Treat **** |  |  |  |  |  |  |
|  | *block ns* |  |  |  |  |  |  |
|  | *G :Treat ** |  |  |  |  |  |  |
| qP |  |  |  |  |  |  |  |
|  | M-WD | 0.50 ± 0.04 | 0.49 ± 0.04 | 0.47 ± 0.06 | 0.46 ± 0.06 | 0.48 ± 0.02 | 0.47 ± 0.02 |
|  | H-WD | 0.43 ± 0.07 | 0.46 ± 0.07 | 0.41 ± 0.10 | 0.43 ± 0.04 | 0.37 ± 0.11 | 0.43 ± 0.08 |
|  | Relative Diff. (%) | -12 | -6 | -13 | -7 | -21 | -10 |
|  | *G *** |  |  |  |  |  |  |
|  | *Treat ns* |  |  |  |  |  |  |
|  | *block ns* |  |  |  |  |  |  |
|  | *G :Treat ns* |  |  |  |  |  |  |
| qN |  |  |  |  |  |  |  |
|  | M-WD | 0.78 ± 0.03 | 0.78 ± 0.06 | 0.84 ± 0.02 | 0.81 ± 0.04 | 0.85 ± 0.03 | 0.84 ± 0.03 |
|  | H-WD | 0.87 ± 0.03 | 0.85 ± 0.06 | 0.86 ± 0.05 | 0.85 ± 0.02 | 0.90 ± 0.02 | 0.88 ± 0.03 |
|  | Relative Diff. (%) | 12 | 8 | 3 | 5 | 6 | 4 |
|  | *G *** | *a* | *a* | *ab* | *ab* | *b* | *ab* |
|  | *Treat **** |  |  |  |  |  |  |
|  | *block ns* |  |  |  |  |  |  |
|  | *G :Treat ns* |  |  |  |  |  |  |

**Table S3.** Correlation values of observed and normalized plant WUE in function of WUEi, in 5 fungus tolerant genotypes and Syrah, Montpellier - FR, 2022.

| Variables |  | Syrah | 3176N | 3159B | Floreal | G14 | G5 | |
| --- | --- | --- | --- | --- | --- | --- | --- | --- |
| WUEpl vs WUEi | | | | | | | |  |
| Correlation | | -0.20 | 0.67 | 0.53 | 0.02 | 0.54 | 0.18 | |
| p.value | | 0.57 | 0.05 | 0.18 | 0.95 | 0.13 | 0.63 | |
|  |  |  |  |  |  |  |  | |
| WUEpl_n vs WUEi | | | | | | | | |
| Correlation | | 0.49 | 0.47 | 0.92 | 0.74 | 0.96 | 0.73 | |
| p.value | | 0.15 | 0.20 | 0.001 | 0.01 | 0.000 | 0.02 | |
|  |  |  |  |  |  |  |  | |


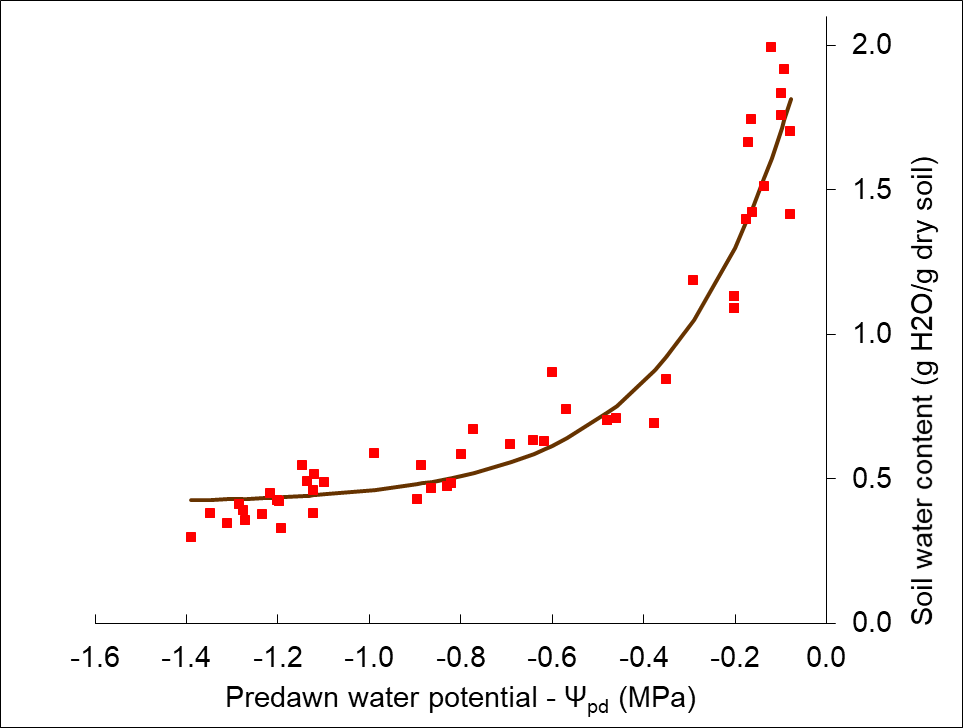


**Figure S1.** water potential relantioship with soil water content (g of water per g of dry soil). Fitted line correspond to Soil water content = a * (exp b* Ψ_pd_) + c, where a, b and c correspond to 1.87, 3.72 and 0.41, respectively.


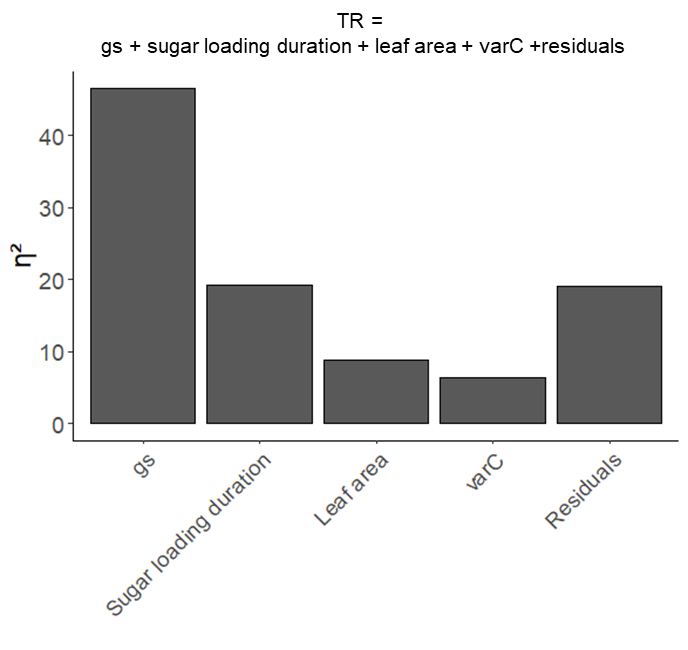


**Figure S2.** Proportion of variance explained by each variable in the multiple linear regression of TR = gs + sugar loading duration + leaf area + varC + residuals, R^2^ = 0.77.


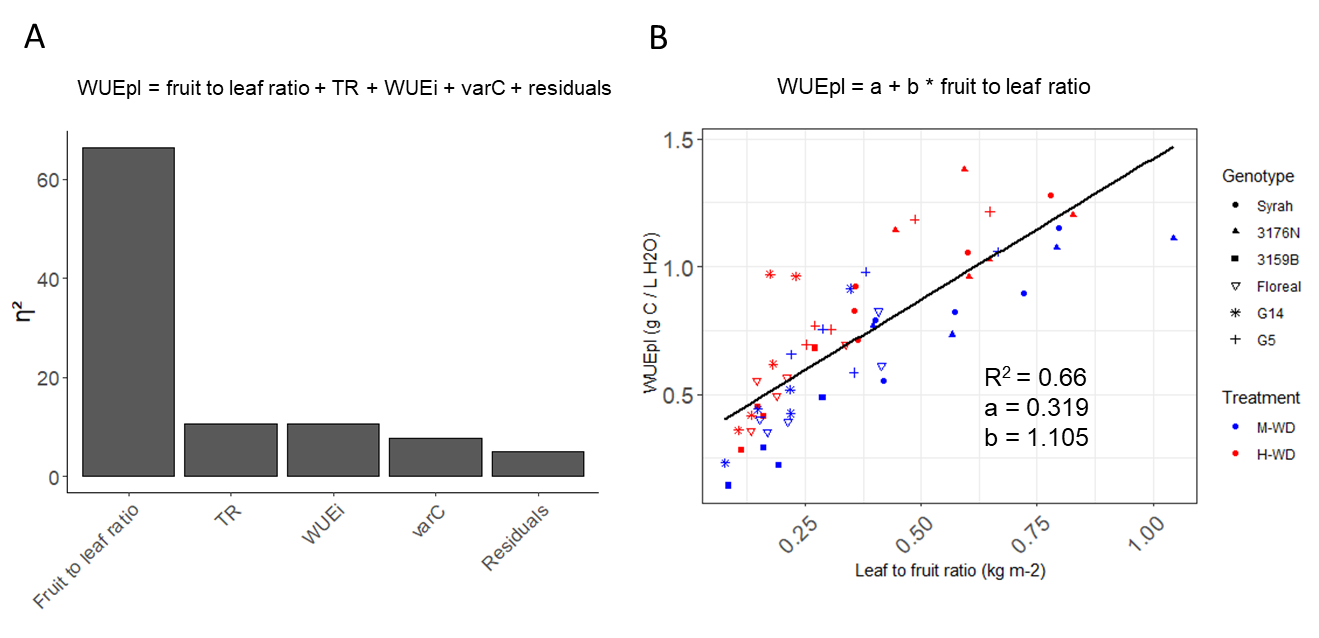


**Figure S3.** Proportion of variance explained by each variable in the multiple linear regression of WUEpl = fruit to leaf ratio + TR + WUEi + varC + residuals, R^2^ = 0.95 (A) and the single linear regression between WUEpl and leaf to fruit ratio.
